# The Genome of the CTG(Ser1) Yeast *Scheffersomyces stipitis* Is Plastic

**DOI:** 10.1128/mBio.01871-21

**Published:** 2021-09-07

**Authors:** Samuel Vega-Estévez, Andrew Armitage, Helen J. Bates, Richard J. Harrison, Alessia Buscaino

**Affiliations:** a University of Kent, School of Biosciences, Kent Fungal Group, Canterbury Kent, United Kingdom; b Natural Resources Institute, University of Greenwich, Chatham Maritime, Kent, United Kingdom; c NIAB Cambridge Crop Research, Cambridge, United Kingdom; Tel Aviv University

**Keywords:** biofuels, CTG clade yeast, genome plasticity, genomic instability, transposons

## Abstract

Microorganisms need to adapt to environmental changes, and genome plasticity can lead to rapid adaptation to hostile environments by increasing genetic diversity. Here, we investigate genome plasticity in the CTG(Ser1) yeast *Scheffersomyces stipitis*, an organism with an enormous potential for second-generation biofuel production. We demonstrate that *S. stipitis* has an intrinsically plastic genome and that different *S. stipitis* isolates have genomes with distinct chromosome organizations. Real-time evolution experiments show that *S. stipitis* genome plasticity is common and rapid since extensive genomic changes with fitness benefits are detected following *in vitro* evolution experiments. Hybrid MinION Nanopore and Illumina genome sequencing identify retrotransposons as major drivers of genome diversity. Indeed, the number and position of retrotransposons are different in different *S. stipitis* isolates, and retrotransposon-rich regions of the genome are sites of chromosome rearrangements. Our findings provide important insights into the adaptation strategies of the CTG(Ser1) yeast clade and have critical implications in the development of second-generation biofuels. These data highlight that genome plasticity is an essential factor for developing sustainable *S. stipitis* platforms for second-generation biofuels production.

## INTRODUCTION

Eukaryotic genomes are often described as stable structures with well-preserved chromosome organization, and genome instability is viewed as harmful. However, an increasing body of evidence demonstrates that eukaryotic microorganisms have a plastic genome and genome instability is instrumental for rapid and reversible adaptation to hostile environments (1–4). This is because genomic instability can increase genetic diversity, allowing the selection of genotype(s) better adapted to a new environment (5, 6). Repetitive DNA elements are major contributors to genome plasticity since repeats can undergo inter- and intralocus recombination, resulting in gene conversion, gross chromosomal rearrangements, and segmental aneuploidies (7). Transposable elements (TEs), a specific class of repetitive elements, alter genome organization by recombination-dependent mechanisms and by jumping to new sites in the genome (8). TEs belong to two major classes: DNA transposons (class II) and retrotransposons (class I). DNA transposons utilize a “cut and paste” mechanism in which the parental element excises from its original location before integrating elsewhere (9). In contrast, retrotransposons replicate through reverse transcription of their RNA and integrate the resulting cDNA into another locus. Retrotransposons can be further classified into long-terminal-repeat (LTR) retrotransposons and non-LTR retrotransposons (10). LTR retrotransposons are characterized by two LTR sequences flanking an internal coding region containing the genes encoding for the structural protein GAG and enzyme POL required for reverse transcription and integration (11). While POL enzymes are conserved across organisms, GAG proteins are poorly conserved (12). LINE elements are one of the most abundant non-LTR retrotransposons, and they are typically composed of a 5′-noncoding region, two open reading frames (ORF1 and ORF2) and a 3′-noncoding region that is marked by a poly(A) tail (13). ORF1 proteins have a diverse amino acid sequence, but they often contain a DNA-binding motif (14). ORF2 encodes endonuclease and reverse transcriptase activity that is critical for transposition (15).

The CTG(Ser1) clade of fungi, in which the CTG codon is translated as serine rather than leucine, is an important group of ascomycetous yeasts featuring those that hold great promise in biotechnology, such as *Scheffersomyces stipitis*, and dangerous human fungal pathogens, such as Candida albicans (16). The CTG(Ser1) clade comprises several species with different lifestyles and genomic organizations, including haploid and diploid species that colonize diverse environments by reproducing sexually or para-sexually (16–19). One common feature of CTG(Ser1) species is their ability to adapt remarkably well to extreme environments (20). For example, CTG(Ser1) yeasts can grow on various carbon sources and are highly tolerant to environmental changes such as changes in osmolarity (16, 19, 20). It is well established that genome plasticity is a critical adaptive mechanism in the human fungal pathogens Candida albicans, the most studied CTG(Ser1) clade member (4). In C. albicans, stress increases genome instability by affecting the rate and type of genomic rearrangements (21). Different classes of DNA repeats drive this genetic variation, including TEs, long repeats and major repeat sequences (MRS) (22–24). It is still unknown whether genome plasticity is a general feature of the CTG(Ser1) clade and whether DNA repeats are drivers for genome diversity across this yeast group.

This study investigates genome plasticity in *S. stipitis*, a CTG(Ser1) clade yeast with great potential for the eco-friendly and ethical production of second-generation biofuels (25–27). Second-generation biofuels are generated by fermentation of lignocellulose biomass, produced in large amounts (>1.3 billion tons produced annually) as waste following agricultural and forestry processing operations (27). Lignocellulose is a heteropolymer composed of fermentable hexose sugars (i.e., glucose) and pentose sugars (i.e., xylose) (28). The yeast Saccharomyces cerevisiae, usually the organism of choice for industrial production of ethanol, is not suitable for producing second-generation ethanol because it cannot ferment pentose sugars as it lacks specific transporters enzymatic network important for their metabolism (28). *S. stipitis* holds excellent potential for biofuel derived from green waste because it is one of the few yeast species that can ferment both hexose and pentose sugars (25–27). *S. stipitis* is a nonpathogenic haploid yeast that is found in the gut of wood-ingesting beetles, in hardwood forests or areas high in agricultural waste (29). Contrary to C. albicans, *S. stipitis* has a canonical sexual cycle whereby mating of haploid cells generate diploid cells that undergo meiosis and produce haploid spores (30). Although several *S. stipitis* natural isolates are used for the optimization of second-generation biofuels production, the genome of only one strain (Y-11545) has been sequenced and assembled to the chromosomal level (31). The Y-11545 genome has a size of 15.4 million base pairs (Mbp) organized in eight chromosomes and containing ∼6,000 protein-coding genes (31–33). *S. stipitis* chromosomes are marked by regional centromeres composed of full-length LTR retrotransposons (Tps5a, Tps5b, and Tps5c) and noncoding, non-autonomous LARD (large retrotransposon derivative) elements (31, 33).

To investigate the plasticity of the *S. stipitis* genome, we have taken several complementary approaches. First, we systematically identified *S. stipitis* DNA repeats and investigated the genotypic diversity of 27 different *S. stipitis* natural isolates collected from different environments. Second, we combined MinION Nanopore with Illumina genome sequencing to generate a high-quality chromosome-level sequence assembly of a second *S. stipitis* natural isolate (Y-7124) and compared its genome structure to the reference Y-11545 genome. Lastly, we performed *in vitro* evolution experiments and analyzed *S. stipitis* genome organization changes following laboratory passaging under stress or unstressed growth conditions. Thanks to this combined approach, we discovered that the *S. stipitis* genome is plastic. We demonstrate that different *S. stipitis* natural isolates have distinct chromosomal organizations and that transposable elements drive this extensive intra-species genetic variation. Genome plasticity is not regulated by stress; however, large chromosome rearrangements are linked to adaptation to hostile environments. Our findings have important implications for second-generation biofuel production as genome plasticity is a paramount factor to be considered for the successful development of superior biofuel-producer *S. stipitis* strains.

## RESULTS

### Classification of *S. stipitis* DNA repeats.

**FIG 1:**
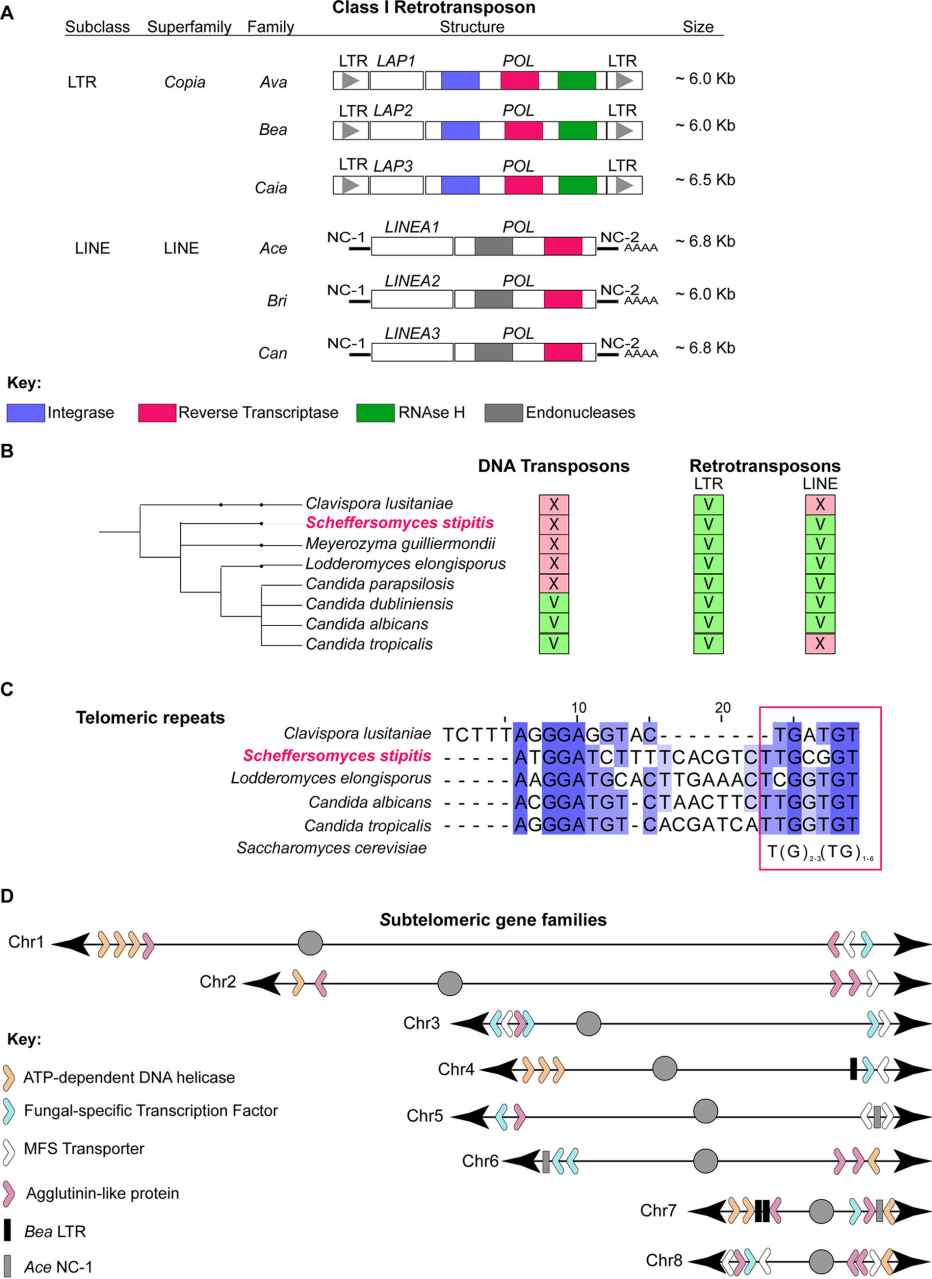
Classification of noncentromeric *S. stipitis* repeats. (A) Schematics of noncentromeric retrotransposons identified in this study. For each transposon, the subclass, superfamily, and family is indicated. The organization of coding and noncoding sequences of each transposon is displayed. (B) Cladogram showing CTG(Ser1) clade species with known transposable elements (this study; see also references 18 and 34). The presence (V) or absence (X) of a TE is indicated. (C) Sequence alignment of telomeric terminal repeats in members of the CTG(Ser1) clade (C. lusitaniae, *S. stipitis*, *L. elongisporus*, C. albicans, and C. tropicalis) (this study; see also references 18 and 34). Consensus sequence to the S. cerevisiae telomeric repeats is indicated (magenta box). (D) Schematics of gene family members associated with *S. stipitis* subtelomeres (30 kb from chromosome end).

DNA repeats are drivers of genome variation. Comparative genomic analyses have identified different repetitive elements in some CTG(Ser1) clade members, yet a comprehensive survey of *S. stipitis* repetitive elements is lacking (18, 34). Therefore, we sought to classify the major classes of repetitive elements associated with the Y-11545 sequenced genome by aligning the genomic sequence of each strain to itself and identifying long sequences (>100 nucleotides [nt]) present more than once in the genome. The genomic position of these repeats was manually verified, and clustered repeats were combined and categorized depending on their genomic positions, structures, and sequence similarity. Our analyses identified known *S. stipitis* repeat-rich loci such as centromeric transposon clusters, the NUPAV sequence, an integrated L-A dsRNA virus, and several gene families (32, 33, 35). As observed in other members of the CTG(Ser1) clade, we did not detect any MRS, a class of repetitive elements found only in C. albicans and the closely related C. dubliniensis and C. tropicalis species (18, 34). Here we focus on intra- or interchromosomal repeats that have not been described to date: noncentromeric TEs, subtelomeric regions, and telomeric repeats (Fig. 1).

We identified six novel retrotransposon families scattered along chromosome arms: three LTR retrotransposons (*Ava*, *Bea*, and *Caia*) and three LINE retrotransposons (*Ace*, *Bri*, and *Can*) (Fig. 1A; see also Table S3 in the supplemental material). *Ava*, *Bea*, and *Caia* have a similar structure where two identical LTR sequences flank an internal domain. The internal domain contains two ORFs: one encoding for a putative POL and one encoding for an *S. stipitis*-specific protein that we named LTR-associated protein (Lap1 in *Ava*, Lap2 in *Bea*, and Lap3 in *Caia*). Homology search failed to identify any GAG gene associated with the *Ava*, *Bea*, and *Caia* retrotransposons. Since Gag proteins are poorly conserved among different organisms, we hypothesize that the Lap proteins are Gag proteins.

10.1128/mBio.01871-21.4 TABLE S3 Noncentromeric retrotransposon associated with the Y-11545 strain. Transposons associated with each chromosome (Chr) are indicated. For each transposon, the subclasses, superfamily, and family are indicated. The retrotransposons position in each chromosome (start and end) and their length (length bp) is shown. For LTR retrotransposons, the following features are shown: right LTR (R-LTR) and left LTR (L-LTR) lengths, the percent sequence identity (LTR identity %) between right and left LTR, the presence of a 5′TG and a 3′CA, the presence of a primer binding site, and the structural domain for ORF1 and POL (integrase reverse transcriptase and RNase H). For LINE retrotransposons, the structural domain of LINEA (LINEA Zn finger) and ORF2 (Endonuclease, Reverse Transcriptase) are indicated. In addition, the presence of a 5′ Poly(A) and 3′ Poly(A) is indicated. Download Table S3, XLSX file, 0.02 MB. Copyright © 2021 Vega-Estévez et al. 2021 Vega-Estévez et al. https://creativecommons.org/licenses/by/4.0/ This content is distributed under the terms of the Creative Commons Attribution 4.0 International license.

*Ace*, *Bri*, and *Can* are LINE elements composed of the noncoding regions NC-1 and NC-2 surrounding an internal coding region encoding for a Pol enzyme and an *S. stipitis*-specific LINE-associated protein (Linea1 in *Ace*, Linea2 in *Bri*, and Linea3 in *Can*). Linea1 and Linea2, but not Linea 3, have a zinc-finger DNA-binding motif (see Table S3). Comparison across the CTG(Ser1) clade revealed that *S. stipitis* TE repertoire is typical of this clade. Indeed, retrotransposons are common in this yeast group: the genome of all species analyzed contains LTR elements, whereas LINE elements are present in 6/8 species (Fig. 1B; see also Table S4). Similarly to other CTG(Ser1) clade yeasts, we did not detect any DNA transposons integrated into the *S. stipitis* genome (Fig. 1B; see also Table S4).

10.1128/mBio.01871-21.5 TABLE S4 Full-length transposons in the CTG(Ser1) clade. For each transposon, the classes, subclasses, family, and systematic name are indicated. Data sources: *S. stipitis* (this study); C. dubliniensis (34); C. albicans SC5314, C. albicans WO-1, C. parapsilosis, *Lodderomyces elongisporus*, C. guilliermondii, and C. lusitaniae (18). Download Table S4, XLSX file, 0.01 MB. Copyright © 2021 Vega-Estévez et al. 2021 Vega-Estévez et al. https://creativecommons.org/licenses/by/4.0/ This content is distributed under the terms of the Creative Commons Attribution 4.0 International license.

Our repeat analysis demonstrates that the terminal sequences of *S. stipitis* chromosomes are repeat-rich and composed of two elements with different degrees of repetitiveness: telomere proximal repeats and subtelomeric regions. The telomeric repeats are noncanonical and composed of 24-nucleotide units repeated in tandem. Each unit contains a TG motif reminiscent of typical telomeric repeats (Fig. 1C). *S. stipitis* subtelomeric regions (the ∼30-kb region adjacent to telomeric repeats) are enriched in retrotransposon-derived elements. Indeed, DNA sequences with homology to *Bea* LTR retrotransposons and *Ace* LINE elements are found in 5/16 subtelomeric regions (Fig. 1D; see also Table S5). No full-length retrotransposons are detected at these genomic locations. Subtelomeric regions contain several gene family members, including gene encoding for ATP-dependent DNA helicases (found in 7/16 subtelomeres), fungus-specific transcription factors (8/16 subtelomeres), MFS transporters (8/16 subtelomeres), and agglutinin-like proteins (11/16 subtelomeres) (Fig. 1D; see also Table S5) (31). In summary, our analysis demonstrates that the *S. stipitis* genome contains several classes of repetitive elements that could be major contributors to genome plasticity.

10.1128/mBio.01871-21.6 TABLE S5 Organization of subtelomeric regions in the Y-11545 strain. Gene families: ORFs located in the 30 kb from chromosome ends are indicated. For each ORF, the systematic names, their genomic locations, and their structural domains are indicated. Transposons: DNA sequences with homology to transposons in the 30 kb from chromosome ends are indicated. For each sequence, the systematic names, their genomic locations, and their structural features are indicated. Download Table S5, XLSX file, 0.02 MB. Copyright © 2021 Vega-Estévez et al. 2021 Vega-Estévez et al. https://creativecommons.org/licenses/by/4.0/ This content is distributed under the terms of the Creative Commons Attribution 4.0 International license.

### *S. stipitis* natural isolates have distinct genomic organizations.

**FIG 2:**
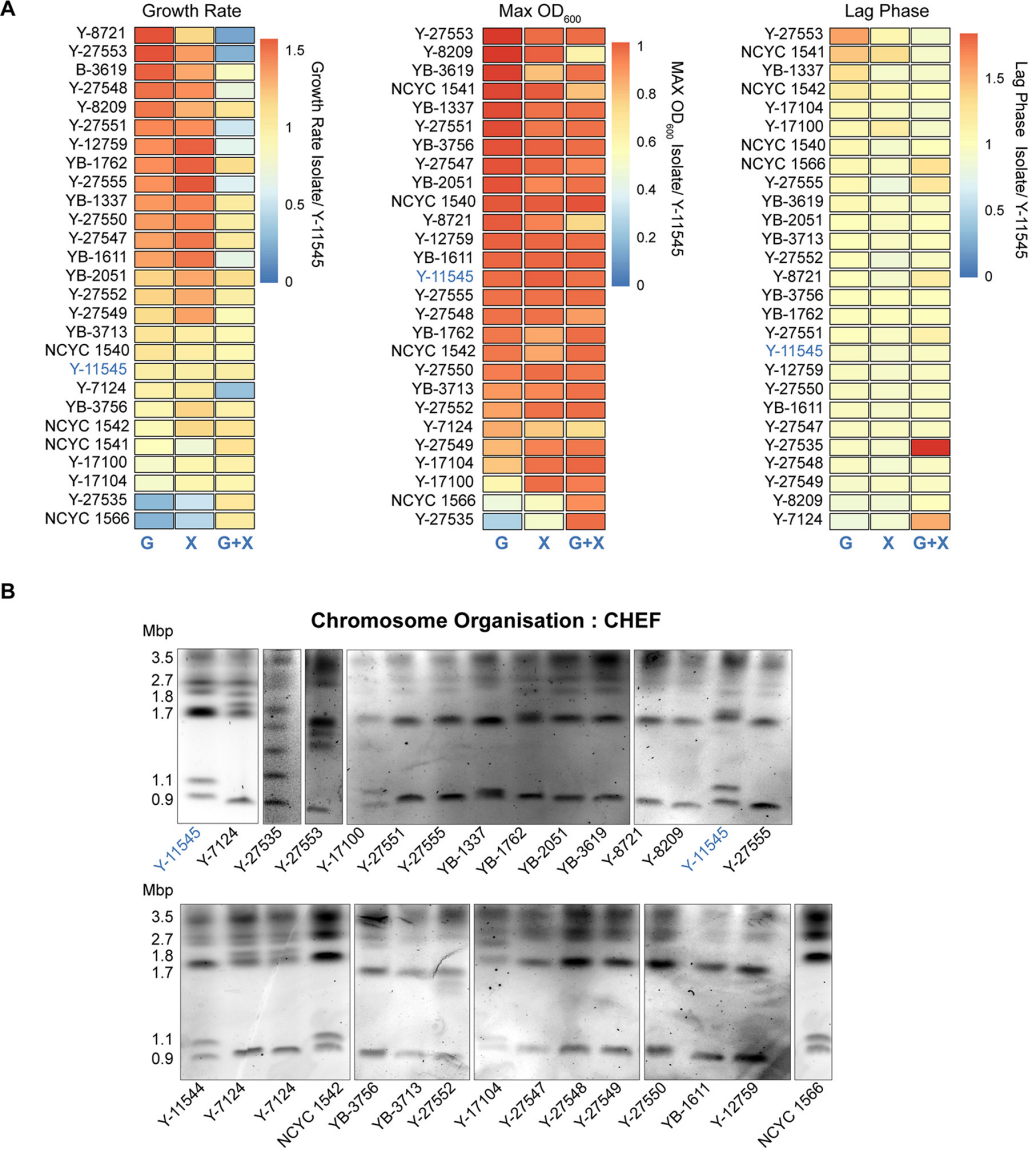
Phenotypic and genotypic diversity in *S. stipitis.* (A) Heatmaps comparing growth rate (left), maximum OD (middle), and lag time (right) for each *S. stipitis* natural isolate in comparison to the reference Y-11545 strain (blue). Analyses were performed in glucose (G), xylose (X), and glucose/xylose (G+X) media. The heatmap data are the average of three biological replicates. (B) Karyotyping of *S. stipitis* natural isolates by CHEF electrophoresis. The Y-11545 strain is highlighted in blue, and the sizes of its eight chromosomes are indicated.

Having identified *S. stipitis* DNA repeats, our next step was to examine *S. stipitis* phenotypic and genotypic diversity across a geographically diverse set of strains (*n* = 27) that were collected in different habitats (see Table S1, source NRRL and NCYC collection) and that include the sequenced Y-11545 strain (31). rDNA fingerprinting confirm that all isolates belong to the *S. stipitis* species (D1/D2 domain of the 26S rDNA similarity >99%) (see Table S6). Phenotypic analyses established that the natural isolates vary in their ability to utilize and grow on different carbon sources. Indeed, compared to the reference Y-11545 strain, different natural isolates cultured in synthetic complete media containing the hexose sugar glucose (SC-G), the pentose sugar xylose (SC-X), or a mixture of both sugars as found in lignocellulose (SC-G+X) display a distinct growth rate, maximum culture density, and lag phase (Fig. 2A; see also Table S7). To determine whether the natural isolates have distinct genomic organizations, we analyzed their karyotype by chromosomes Contour-clamped Homogenous Electric Field (CHEF) gel electrophoresis, a technique allowing chromosome separation according to size. The CHEF electrophoresis analysis reveals clear differences in chromosome patterns demonstrating that *S. stipitis* natural isolates have a genome organized in different-sized chromosomes (Fig. 2B). We concluded that intraspecies phenotypic and genotypic variation is a common feature of *S. stipitis*.

10.1128/mBio.01871-21.2 TABLE S1 Strains used in this study. Strains were obtained from two public depositories (Collection): NRRL and NCYC. For each strain, the systematic name (identifier), collection habitat (habitat), region (region), and country (country) of origin are indicated. Download Table S1, DOCX file, 0.02 MB. Copyright © 2021 Vega-Estévez et al. 2021 Vega-Estévez et al. https://creativecommons.org/licenses/by/4.0/ This content is distributed under the terms of the Creative Commons Attribution 4.0 International license.

10.1128/mBio.01871-21.7 TABLE S6 Identification of *S. stipitis* natural isolates by Sanger sequencing of the D1/D2 domain of the 26S rDNA gene. Strain name homology to *S. stipitis* (% identity) and the expected value (E) are indicated. Download Table S6, DOCX file, 0.01 MB. Copyright © 2021 Vega-Estévez et al. 2021 Vega-Estévez et al. https://creativecommons.org/licenses/by/4.0/ This content is distributed under the terms of the Creative Commons Attribution 4.0 International license.

10.1128/mBio.01871-21.8 TABLE S7 Statistical differences in growth rate, maximum OD, and lag times of each natural isolate used in this study compared to the reference strain Y-11545. The significant differences for each strain grown on SC-glucose (SC-G), SC-xylose (SC-X), and SC-60% glucose, 40% xylose (SC-G+X) are indicated (***, **, and *). Download Table S7, DOCX file, 0.02 MB. Copyright © 2021 Vega-Estévez et al. 2021 Vega-Estévez et al. https://creativecommons.org/licenses/by/4.0/ This content is distributed under the terms of the Creative Commons Attribution 4.0 International license.

### Hybrid genomic sequencing identifies transposable elements as drivers of *S. stipitis* genome plasticity.

To date, only one *S. stipitis* isolate (Y-11545) has been sequenced and assembled at chromosome level (31). To gain insights into *S. stipitis* genetic diversity, we generated a chromosome-level sequence assembly of a second *S. stipitis* natural isolate (Y-7124) by combining MinION Nanopore with Illumina genome sequencing. This isolate was chosen because (i) karyotypic analysis reveals that its genomic organization is distinct from the genomic organization of the reference strain Y-11545 and (ii) Y-7124 is widely used both for industrial applications and for basic research (36).

**FIG 3:**
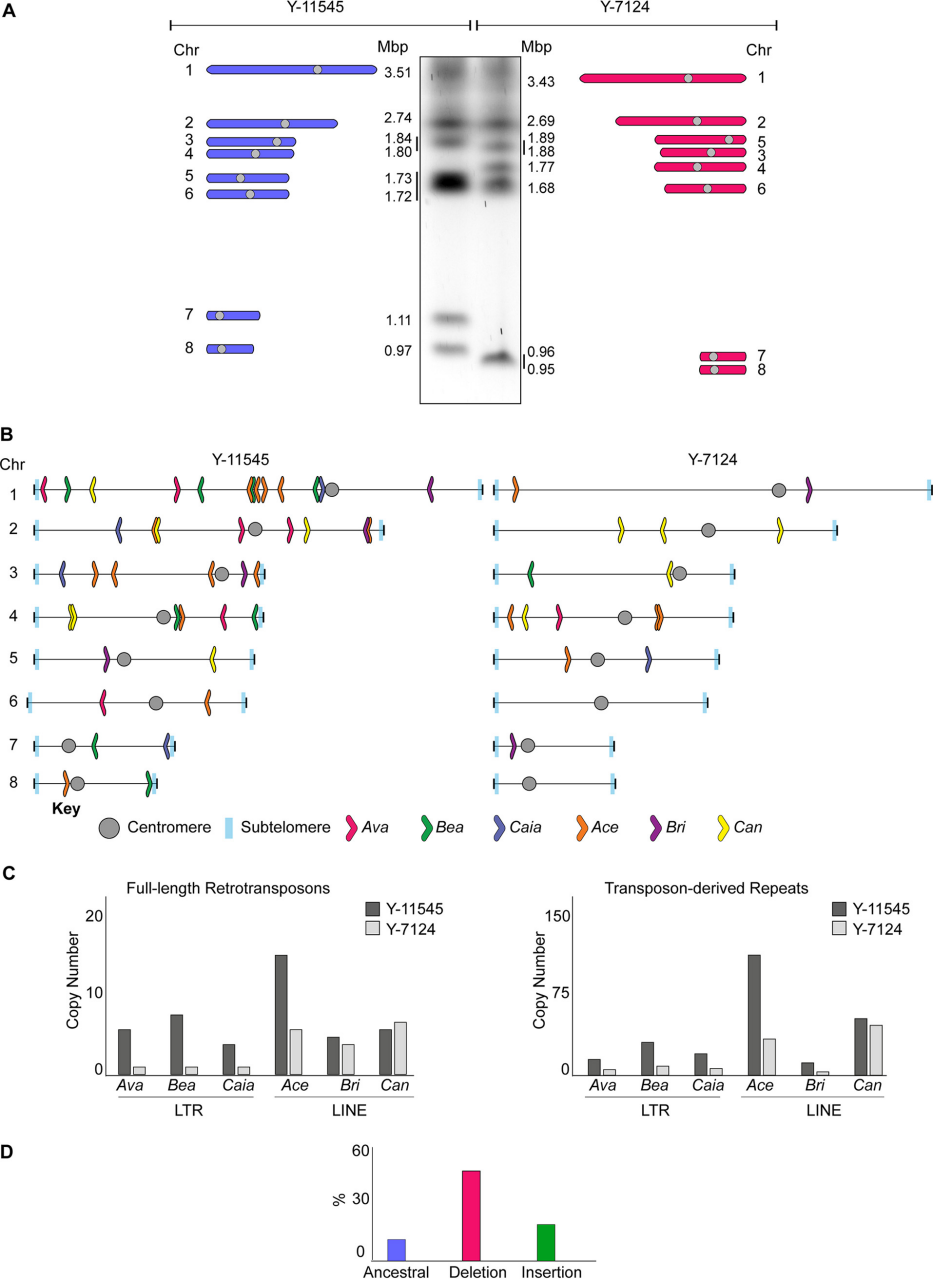
Differences in TE distribution and organization. (A) The genomic organization of Y-11545 and Y-7124 is distinct. (Left) Schematics of Y-11545 chromosome organization. The chromosome (Chr) number and size (Mbp) are indicated. (Middle) Karyotyping of *S. stipitis* Y-11545 and Y-7124 strains by CHEF electrophoresis. (Right) Schematics of Y-7124 chromosome organization. The chromosome (Chr) number and size (Mbp) are indicated. (B) Schematics of noncentromeric transposon family distribution in Y-11545 (left) and Y-7124 (right). (C) Copy numbers of full-length transposons (Left) and transposon-associated repeats associated with the Y-11545 (dark gray) and Y-7124 (light gray) genome. (D) Percentages (%) of ancestral, deletion, and insertion sites associated with the Y-11545 and Y-7124 genomes.

The Y-7124 genome was sequenced to 186.88× coverage resulting in a 15.69-Mb assembly arranged in 10 contigs (see Table S8). High-accuracy reads from Illumina sequencing enabled the correction of errors that are associated with the MinION technology. A final chromosome-level assembly was produced by manually identifying overlapping regions between contigs. Comparing the Y-7124 and Y-11545 nucleotide sequences reveals that the two natural isolates overall share a similar coding DNA sequence. The total number of single-nucleotide polymorphisms (SNPs) between the two natural isolates is 50,495, equating to one variant every 306 bases. The majority of these SNPs are synonymous changes (16,294 = 74.25%), while ∼25% (5,622) of the SNPs are missense, and only 0.13% (28) are nonsense (see Table S9). Despite this high DNA sequence similarity, the Y-7124 genome is organized in eight chromosomes with different sizes and organizations from that of Y-11545 (Fig. 3A). Comparison of the Y-7124 and Y-11545 genomes establishes that retrotransposons are significant drivers of *S. stipitis* genome diversity as one of the most prominent differences between the two genomes is the abundance and localization of these retrotransposons (Fig. 3B). Indeed, the number of LTR and LINE noncentromeric retrotransposons and transposon-derived repeats is greater in the Y-11545 reference genome compared to the Y-7124 genome: retrotransposons, solo LTRs, and truncated LINE elements account for approximately 2% of the reference Y-11545 genome and only for ∼1% of the Y-7124 genome (Fig. 3C). We classified retrotransposons loci present in both isolates (ancestral loci), those present in the reference Y-11545 genome but absent in Y-7124 (deletion loci), and those not present in the reference genome but present in a given strain (insertion loci). Of 69 transposon loci, only 10 ancestral loci (∼15%) were detected in the two isolates. These sites are likely to be inactive transposons or transposons that rarely transpose. In addition, we detected 42 deletion loci (60%) and 17 (24%) insertion loci (Fig. 3D). The presence of deletion and insertion loci suggests that *S. stipitis* LTR transposons and LINE elements are active and competent for transposition. Although active transposons can insert into genes to cause functional consequences (37), we did not detect any TE-driven alteration in coding regions.

10.1128/mBio.01871-21.9 TABLE S8 Quality assessment of the Y-7124 genome sequencing. (Top) Number of contigs of the final assembly, including the total length (Mbp) and the size of the largest contig (Mbp). N50: total length of the shortest contig at 50% of the genome length (50% of the genome is contained in contigs larger than this value). L50: smallest number of contigs whose length sum up makes half of the genome size. (Bottom) Busco analysis outcome using the Saccharomycetales_odb9 gene database containing 1,711 genes. The numbers of unique (complete), duplicated, and fragmented and missing genes are indicated. Download Table S8, DOCX file, 0.01 MB. Copyright © 2021 Vega-Estévez et al. 2021 Vega-Estévez et al. https://creativecommons.org/licenses/by/4.0/ This content is distributed under the terms of the Creative Commons Attribution 4.0 International license.

10.1128/mBio.01871-21.10 TABLE S9 Summary of the SNPs observed between the natural isolates of *S. stipitis* NRRL Y-11545 and NRRL Y-7124. The table shows the number of variants, the variant rate, and the number of transitions and transversions. The numbers and relative percentages of SNPs occurring in exon and intergenic regions is shown. The numbers and relative percentages of SNPs causing missense, nonsense, and silent mutations are indicated. Download Table S9, DOCX file, 0.01 MB. Copyright © 2021 Vega-Estévez et al. 2021 Vega-Estévez et al. https://creativecommons.org/licenses/by/4.0/ This content is distributed under the terms of the Creative Commons Attribution 4.0 International license.

### Transposable elements are sites of chromosome rearrangements.

**FIG 4:**
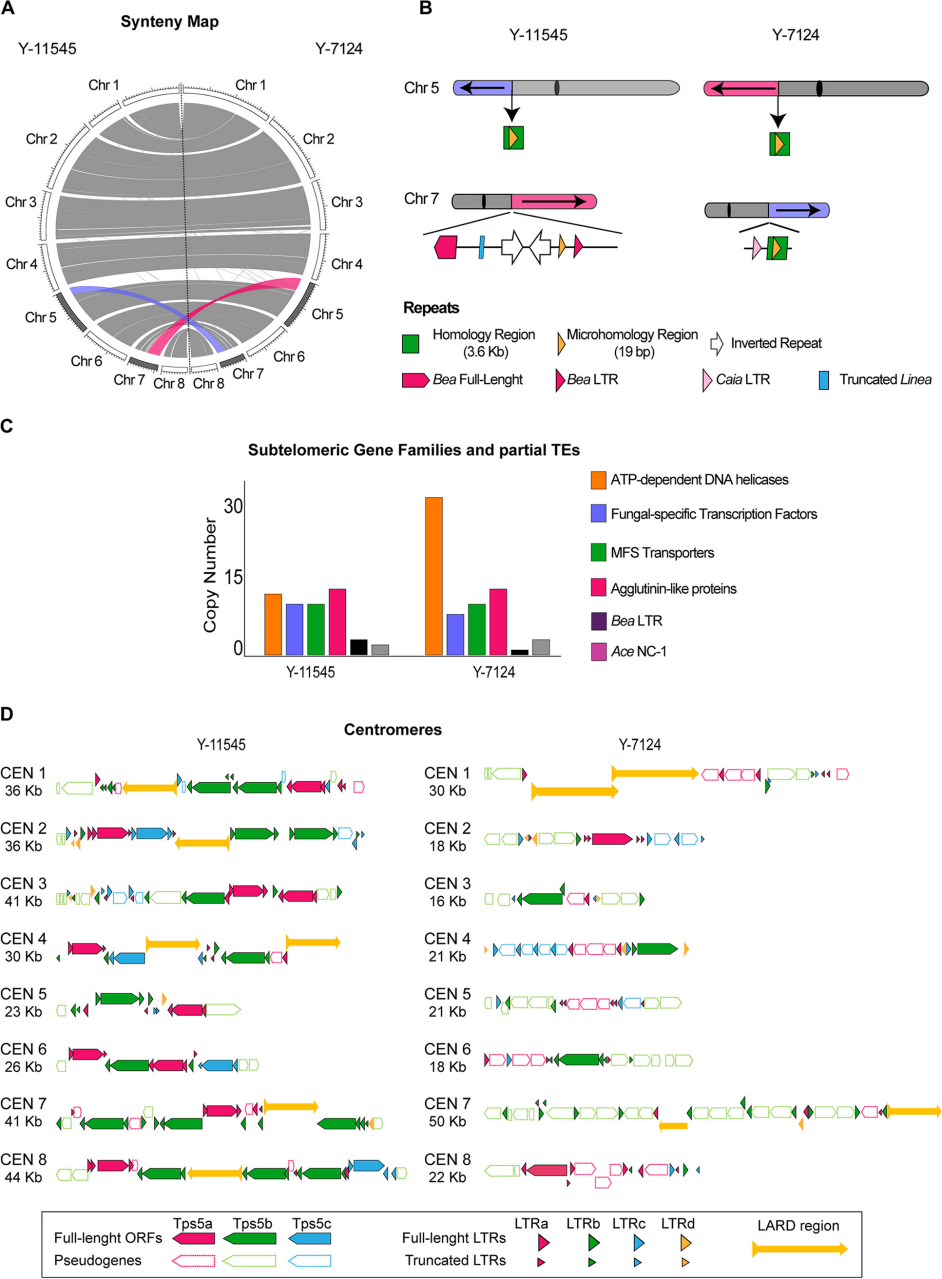
Chromosome rearrangements between *S. stipitis* natural isolates. (A) Circos plot displaying macrosynteny between Y-11545 (left) and Y-7124 (right). Chromosome (Chr) number and size is indicated. Reciprocal translocation between the two genomes is highlighted in purple and pink. (B) Schematics of repetitive sequences associated with the translocation junction in the Y-11545 (left) and Y-7124 (right) genomes. (C) Subtelomeric gene families and TEs distribution in the Y-11545 and Y-7124 genomes. (D) Schematics of centromere organization in the Y-11545 (left) and Y-7124 (right).

Comparison of the Y-11545 and Y-7124 genome reveals that transposon-rich regions are sites of complex chromosome rearrangements since a transposon-rich region is the breakpoint of a reciprocal translocation between chromosome 5 and chromosome 7. Indeed, chromosome 5^Y-7124^ (Chr5^Y-7124^) comprises two parts: one corresponding to Chr7^Y-11545^ (pink) and one corresponding to Chr5^Y-11545^ (gray). Likewise, Chr7^Y-7124^ contains sequences corresponding to Chr5^Y-11545^ (purple) and one corresponding to Chr7^Y-11545^ (gray) (Fig. 4A and B).

This translocation causes the size change of Chr5^Y-7124^ and Chr7^Y-7124^ detected by CHEF karyotyping (Fig. 4A). Southern analyses with a probe specific for Chr5^Y-11545^ (nt 448855 to 449034) confirms that this genomic sequence is associated with the smaller chromosome of ∼0.96 Mbp (Chr7 size) in strain Y-7124 (see Fig. S1). The evolutionary history of Y-11545 and Y-7124 is unknown, and therefore it is difficult to predict the molecular events underlying these genomic changes. However, sequence analysis of the rearrangement breakpoint reveals that this structural variation occurs in a genomic region that (i) contains homologous sequences between chromosomes 5 and 7 and (ii) is transposon-rich and contains two inverted repeats on chromosome 7 (Fig. 4B). A second significant difference between the genome organization of Y-11545 and Y-7124 is found at subtelomeric regions: these regions differ in the number and organization of subtelomeric gene families and in the number of transposon-associated repeats (Fig. 4C). Lastly, we detected a distinct centromeres organization where the numbers of *Tps5* retrotransposons, LTRs and LARD regions differ between the two isolates (Fig. 4D). The presence of transposons and transposon-derived repeats associated with all these genomic locations strongly suggest that retrotransposons have mediated the chromosomal rearrangement by recombination-mediated mechanisms. Therefore, changes in transposons organization are responsible for the bulk of genomic changes identified in two different natural isolates.

10.1128/mBio.01871-21.1 FIG S1 Southern blot analysis confirm the Chr5/Chr7 translocation. (Left) Schematics of chromosome 5 (Chr5) and chromosome 7 (Chr7) in Y-11545 (left) and Y-7124 (right). Reciprocal translocation is highlighted in purple and pink. A Southern probe is indicated. (Right) Southern blot of Y-11545 and Y-7124 chromosomes separated by CHEF gel electrophoresis. Full chromosome profiling (EtBr) and Southern blot results (Southern) are indicated. Download FIG S1, TIF file, 1.5 MB. Copyright © 2021 Vega-Estévez et al. 2021 Vega-Estévez et al. https://creativecommons.org/licenses/by/4.0/ This content is distributed under the terms of the Creative Commons Attribution 4.0 International license.

### *S. stipitis* real-time evolution leads to extensive genomic changes.

**FIG 5:**
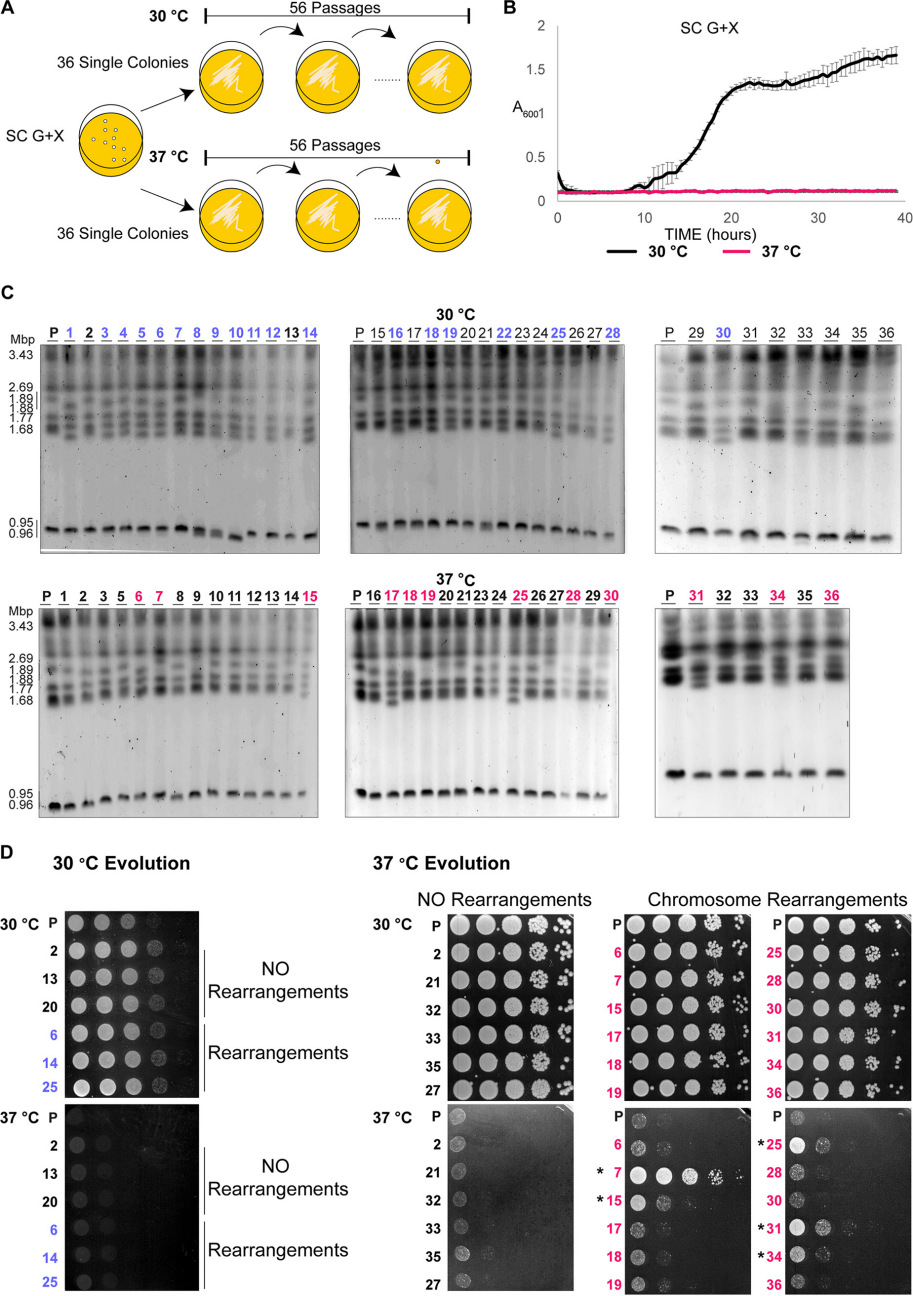
*In vitro* evolution induces *S. stipitis* genome plasticity. (A) Schematics of laboratory evolution strategy. (B) *S. stipitis* growth curve in SC G+X liquid media at permissive (30°C) and restrictive (37°C) temperature. (C) Karyotype organization of *S. stipitis* colonies after 8 weeks of laboratory evolution at 30 and 37°C. Gross chromosomal rearrangements following laboratory evolution at 30°C (blue) and 37°C (magenta) are indicated. The low resolution of CHEF detects changes in smaller chromosomes more precisely than changes in larger chromosomes. (D) Serial dilution assay showing growth at 30 and 37°C of the following strains: parental (P), 30°C-evolved strains without (NO Rearrangements) or with (Rearrangements) and 37°C-evolved strains without (NO Rearrangements) or with (Rearrangements). The CHEF analysis strain number is indicated. *, Colonies with a fitness advantage compared to the parental strain.

Our results demonstrate that intraspecies genetic diversity is common in *S. stipitis*. However, since the evolutionary history of the analyzed natural isolates is unknown, it is difficult to predict whether the observed genomic changes are due to the selection of rare genomic rearrangements events. To determine the time scale of *S. stipitis* genome evolution, we investigated the genome organization of 72 single colonies passaged daily for 8 weeks (56 passages, ∼672 divisions) in SC-G+X, since its sugar composition resembles what found in lignocellulose (29) (Fig. 5A). Strains were grown at 30°C, a temperature that does not lead to any growth defect, and 37°C, a stressful temperature that strongly inhibits *S. stipits* growth (Fig. 5B). CHEF gel electrophoresis was conducted to identify possible changes in the chromosome organization of the evolved strains. This analysis identifies genome rearrangements in 19/36 strains evolved at 30°C and 12/36 strains evolved at 37°C (blue and magenta, Fig. 5C). Thus, changes in chromosome organization were detected in the presence (37°C) or absence (30°C) of stress. To test whether chromosome rearrangements are associated with a fitness benefit, we tested the ability of the parental, 30°C-evolved, and 37°C-evolved strains to grow in SC-G+X media at permissive (30°C) and restrictive (37°C) temperatures (Fig. 5D). This analysis demonstrates that 30°C-evolved strains, with or without chromosomal rearrangements, do not grow well at the restrictive temperature (Fig. 5D). Similarly, 37°C-evolved strains with no chromosomal rearrangement grow poorly at 37°C (Fig. 5D). In contrast, 5/12 37°C-evolved strains with chromosome rearrangements grow better than the parental strain at this restrictive temperature (Fig. 5D). This result suggests that changes in chromosome organization have an adaptive value. Thus, genome plasticity is a defining feature of the *S. stipitis* genome, and its genome can rapidly change in mitotic cells propagated *in vitro*. Our results strongly suggest that the extensive genomic changes can lead to adaptation to hostile environments.

## DISCUSSION

We demonstrate here that the yeast *S. stipitis* has a plastic genome and that genome plasticity is linked to adaptation to hostile environments. We show that noncentromeric retrotransposons are significant drivers of *S. stipitis* genome diversity. These findings have important implications for developing economically viable second-generation biofuels and better understanding the CTG(Ser1) clade biology.

### Retrotransposon are drivers of *S. stipitis* genome diversity.

Our repetitive sequence analysis demonstrates that *S. stipitis* has a DNA repeat content typical of the CTG(Ser1) clade, including TEs, noncanonical terminal telomeric repeats, and subtelomeric regions. As observed in other members of the CTG(Ser1) clade (18), we did not detect any DNA transposons or MRS.

One of our major findings is that that noncentromeric retrotransposons are significant drivers of *S. stipitis* genome diversity. Our data support the hypothesis that *S. stipitis* TEs generate genome diversity via two distinct mechanisms: transposition into new genomic locations and recombination-mediated chromosome rearrangements. Indeed, we demonstrated that the number and genomic position of noncentromeric retrotransposons vary between the Y-11545 and Y-7124 *S. stipitis* isolates. Significantly, we did not detect transposon insertions into coding regions. However, transposons might alter *S. stipitis* gene expression by inserting into gene regulatory regions (37). We propose that *S. stipitis* transposons are active and generate genome diversity by jumping into different genomic locations. Our data also indicate that TEs can generate further genome diversity through either homologous recombination of nearly identical TE copies or by faulty repair of double-strand breaks generated during transposable elements excision (37). Indeed, we find that the translocation breakpoint between chromosome 5 and chromosome 7 is enriched in retrotransposons. Furthermore, TE-rich subtelomeric regions and centromeres have a distinct organization in the two analyzed isolates, suggesting that the transposons drive this genetic diversity. We hypothesize that transposons elements cause the genetic variability observed during laboratory passaging. In the future, it will be important to combine the hybrid genome sequencing approaches and RNA sequencing to identify the underlying genomic and transcriptional changes.

### Genome plasticity and production of second-generation biofuels.

One of our key findings is that the *S. stipitis* genome is intrinsically plastic and that chromosome rearrangements are frequent events under stress or unstressed conditions. Second-generation biofuels, generated by fermentation of agriculture and forestry waste, have an enormous potential to meet future energy demands and significantly reduce petroleum consumption. To meet the requirements for industrial applications, second-generation biofuels need to be generated by microorganisms that can efficiently utilize and ferment all the sugars found in lignobiomass (38). Consequently, *S. stipitis* is one of the most promising yeasts for producing second-generation bioethanol since it can efficiently ferment both hexose and pentose sugars (25, 26, 29). However, robust economically viable *S. stipitis* platforms still require significant development as this organism struggles to survive under the harsh environments generated during second-generation biofuel production. For example, *S. stipitis* growth and fermentation is inhibited by the chemical pretreatment required to extract glucose and xylose from lignobiomass (36). Growth is also inhibited at high ethanol concentrations, and *S. stipitis* ferments xylose less efficiently than glucose. Evolutionary engineering approaches under selective conditions (i.e., the presence of inhibitory compounds, high concentrations of xylose or ethanol) have been applied to isolate better-performing *S. stipitis* strains (36).

Our data predict that the genetic makeup and associated improved phenotypes of superior biofuel producer strains are unstable and that the genetic drivers of improved phenotypes might be lost over time. This hypothesis could explain why short-read Illumina genome sequencing has failed to identify point mutations or indels that could explain the superior performance of *S. stipitis* strains (39). It is also possible that *S. stipitis* superior strains carry stable complex chromosomal rearrangements with a breakpoint at DNA repeats. Such rearrangements could not have been identified by Illumina sequencing since short-sequenced fragments will not resolve changes associated with long repetitive elements. Thus, economically viable use of *S. stipitis* for second-generation biofuels production will require an in-depth analysis of the genomic structures of superior strains.

### Genome plasticity in the CTG(Ser1) clade.

The CTG(Ser1) clade is an incredibly diverse yeast group that includes many important human pathogens and nonpathogenic species (17). Our data support the hypothesis that genome plasticity is a general feature of the CTG(Ser1) yeast clade as it has been observed in C. albicans and *S. stipitis* (21, 22, 40; this study), two organisms with very different lifestyle. Indeed, while C. albicans is a diploid opportunistic human fungal pathogen that lives almost exclusively in the human host; *S. stipitis* is a haploid nonpathogenic yeast found in the gut of wood-ingesting beetles hardwood forests or areas high in agricultural waste (29, 41). Furthermore, while C. albicans lacks a canonical sexual cycle and its associated meiosis, *S. stipitis* has a canonical sexual cycle whereby mating of haploid cells generate diploid cells that undergo meiosis and produce haploid spores (30).

Our results highlight that stress might regulate genome plasticity differently in C. albicans and *S. stipitis*. It has been demonstrated that stress exacerbates C. albicans genome instability (21, 42). In contrast, results presented in this study suggest that *S. stipitis* genome instability is not regulated by stress since we detected a similar rate of chromosomal rearrangements when cells are continuously passaged in unstress (30°C) or stress (37°C) conditions. Importantly, we also demonstrated that the large genomic changes are associated with fitness benefits suggesting that genome plasticity is instrumental for adaptation to hostile environments.

In summary, our study demonstrates for the first time that *S. stipitis* genome is plastic. Understanding the cause and effect of this extensive genome plasticity is paramount to understanding the biology of the CTG(Ser1) clade of fungi.

## MATERIALS AND METHODS

### Yeast strains and growth conditions.

Strains were obtained from the Agricultural Research Service (ARS) Collection, run by the Northern Regional Research Laboratory (NRRL; Peoria, IL), or the National Collection of Yeast Cultures (NCYC; Norwich, United Kingdom) (see Table S1) and confirmed by sequencing (primers AB798 and AB799 of the 26S rDNA [D1/D2 domain]) (43) (see Table S2). Routine culturing was performed at 30°C with 200-rpm agitation on yeast extract/peptone/d-glucose (YPD) media. Phenotypic and *in vitro* evolution analyses were conducted on synthetic complete (SC) media containing glucose (SC-G), xylose (SC-X), or a mixture of 60% glucose and 40% xylose (SC-G+X). SC-G was used as a reference medium since glucose is the preferred carbon source for both the model system S. cerevisiae and *S. stipitis*, SC-X was used because of *S. stipitis* unique ability to utilize xylose as a carbon source, and SC-G+X was used because this sugar combination resembles the ratio found in lignocellulose (28). Uridine (0.08 g/liter in YPD and SC) and adenine hemisulfate (0.05 g/liter in YPD) were added as growth supplements. Solid media were prepared by adding 2% agar.

10.1128/mBio.01871-21.3 TABLE S2 Primers used in this study. For each primer, the systematic name (NAME) and DNA sequence (SEQUENCE) is indicated. Download Table S2, DOCX file, 0.01 MB. Copyright © 2021 Vega-Estévez et al. 2021 Vega-Estévez et al. https://creativecommons.org/licenses/by/4.0/ This content is distributed under the terms of the Creative Commons Attribution 4.0 International license.

### CHEF electrophoresis.

Intact yeast chromosomal DNA was prepared as previously described (44). Briefly, cells were grown overnight, and spheroplasts were prepared in an agarose plug by treating cells (OD_600_ = ∼7) with 0.6 mg/ml Zymolyase 100T (Amsbio, catalog no. 120493-1) in 1% Low-Melt agarose (Bio-Rad, catalog no. 1613112). Chromosomes were separated in a 1% Megabase agarose gel (Bio-Rad) in 0.5× TBE using a CHEF DRII apparatus. Run conditions were as follows: 60- to 120-s switch at 6 V/cm for 12 h, followed by a 120- to 300-s switch at 4.5 V/cm for 12 h at 14°C. Chromosomes were visualized by staining the 0.5× TBE gel with ethidium bromide (0.5 μg/ml) for 30 min, followed by destaining in water for 30 min. Images were capture using a Syngene GBox Chemi XX6 gel imaging system.

### Southern blotting.

DNA from CHEF gels were transferred overnight to a Zeta-Probe GT Membrane (Bio-Rad, catalog no. 162-0196) in 20× SSC and cross-linked using UV (150 mJ). Probing and detection of the DNA were conducted as previously described (45). Briefly, probes were generated by PCR incorporation of DIG-11-dUTP into target sequences according to the manufacturer’s instructions (Roche). Chromosome 5-to-chromosome 7 translocation was detected using a probe generated with the primers AB1028 and AB1029. The probe hybridized to two different chromosomes: in strain Y-11545, the probe hybridized with Chr5 (nt 448855 to 449034), and in strain Y-7124, the probe hybridized with Chr7 (nt 494698 to 494877) (see Table S2). The membrane was incubated with the probe overnight at 42°C with DIG Easy Hyb (Roche, catalog no. 11603558001). The DNA was detected with anti-digoxigenin-alkaline phosphatase antibody (Roche, catalog no. 11093274910) and CDP Star Ready-to-Use (Roche, catalog no. 12041677001) according to the manufacturers’ instructions.

### Phenotypic characterization.

Growth analyses were performed using a plate reader (SpectrostarNano; BMG Labtech) in a 96-well plate format at 30°C for 48 h in SC-G, SC-X, or SC-G+X. The growth rate (μ, h^−1^) was calculated using: μ = [ln(*X*_2_) – ln(*X*_1_)]/(*t*_2_ – *t*_1_), where *X*_1_ is the biomass concentration (OD_600_) at time point 1 (*t*_1_, lag time), and *X*_2_ is the biomass concentration (OD_600_) at time point 2 (*t*_2_, end of exponential growth phase). The maximum OD (OD units) was determined using MAX() from Excel (Microsoft). The lag time (in minutes) was determined visually as the time within which the exponential growth starts. Experiments were performed in three technical and three biological replicates. Heatmaps showing the average of three biological replicates were generated by R using the library *pheatmap*. Analysis of variance was performed to study differences in growth rate, maximum OD, and lag time between the strains. The equality of variances presumption was tested using Levene’s test, whereas the Shapiro-Wilk test was used to assess the normality of the data. When both assumptions were satisfied, a Tukey’s honest significant test was used to determine significant differences between the natural isolates and the reference Y-11545 strain. When the assumption of equal variance was violated, a one-way test was used to indicate significance. In the case of equal variances but a nonnormal distribution of data, the Kruskal-Wallis rank sum test was used to indicate statistical differences, and significance was determined by pairwise testing. A *P* value lower than 0.05 was considered significant for all these statistical tests. Statistical tests were performed using R.

### *In vitro* laboratory evolution.

A single colony of the *S. stipitis* strain NRRL Y-7124 was grown overnight in 5 ml of YPD at 30°C, plated in YPD at a cell density of 100, and grown 48 h at 30°C. A total of 36 single colonies were streaked in two SC-G+X plates and grown at 30 and 37°C, respectively, and streaked daily for a total of 56 passages (8 weeks). The karyotype variability of the colonies was assessed by CHEF electrophoresis. Phenotypic differences were assessed by spotting assays. Strains with rearrangements were grown overnight in SC-G+X and diluted to an OD_600_ of 1. From this, five 1/10 serial dilutions were prepared, and the cells were plated in SC-G+X using a replica plater (Sigma-Aldrich, R2383-1EA) and grown for 48 h at both 30 and 37°C. Strains with no karyotypic modifications after evolution were also used as controls.

### Identification of DNA repeats.

Long sequences (>100 nt) present more than once in the Y-11545 and Y-7124 genomes were identified by aligning each genome to itself using BLASTN. Repetitive elements (E < 1e–04) were manually verified using IGV/SNAPGene, and clustered repeats were combined. This repeat data set was manually examined to further classify it as (i) related to transposable elements, (ii) telomeric repeats, (iii) centromeres, (iv) belonging to protein-coding gene families, and (v) MRS. Transposons were classified using established guidelines (10). Briefly, LTR transposons were identified by detecting two LTR sequences (size, 260 to 430 nt) flanking an internal coding region. These potential LTR transposons were further annotated for the presence of the following marks: an LTR flanked by a TG and CA dinucleotides, the presence of a primer binding site with homology to *S. stipitis* tRNAs (GtRNAdb [http://gtrnadb.ucsc.edu/index.html]), the presence of a coding region with homology to *pol* gene and containing an integrase (INT), reverse transcriptase (RT), and an RNase H (RH) domain. Non-LTR LINE transposons were identified by detecting coding regions homologous to LINE retrotransposons ORF1 (containing a Zn-finger) and ORF2 (containing an endonuclease and a reverse transcriptase domain) and a terminal poly(A) sequence. Retrotransposons were classified into different families based on sequence similarity with a 90% cutoff. Terminal telomeric tandem repeats were identified using Tandem Repeats Finder (46) with default parameters. Regional centromeres were identified based on them being the only regions of the genome with a large retrotransposon Tps5 cluster (ca. 20 to 40 kb) as previously described (33). Gene families were identified by extracting coding regions from our repeat data sets and performing Clustal Omega sequence alignment and PFAMs domain identification using SMART (http://smart.embl.de) (47). The identified gene families were compared to published information (31). The presence of MRS was explored using BLASTN and by searching for clusters of noncoding tandem repeats, a hallmark of C. albicans MRS, with no homology to retrotransposons and not located at chromosome ends. Sequence alignments were visualized with Jalview v2.11.1.0 (48). Phylogenetic trees were generated with phyloT, a phylogenetic tree generator (biobyte.de) using default parameters, and visualized with Itol (https://itol.embl.de/).

### Genome sequencing.

The genome of *S. stipitis* isolate Y-7124 was sequenced by Illumina short-read and MinION long-read technologies. To this end, DNA was extracted from an overnight culture using a Qiagen genomic tip 100/G kit (Qiagen, catalog no. 10243) according to manufacturer’s protocol. For long-read sequencing, MinION (Oxford Nanopore, Oxford, UK) was performed on a DNA library prepared from size-selected gDNA. DNA fragments greater than 30 kb were selected using a Blue Pippin (Sage Science) and concentrated using AmPure beads. From this, a DNA library was prepared using a 1D ligation sequencing kit (SQK-LSK108) and run on the Oxford Nanopore MinION flow-cell FLOMIN 106D. The same gDNA extract was also used for the preparation of Illumina libraries. In this case, the DNA was sheared using a Covaris M220 with microTUBE-50 (catalog no. 520166) and size selected using the Blue Pippin (Sage Science). The library was constructed using a PCR-free kit with NEBNext End Repair (E6050S), NEBNext dA-tailing (E6053S), and Blunt T/A ligase (M0367S) New England Biolabs modules. Sequencing was performed on a MiSeq benchtop analyzer (Illumina) using a 2×300-bp PE (MS-102-3003) flow cell.

### Genome assembly.

Base-calling and demultiplexing were conducted with Albacore v2.3.3 (https://community.nanoporetech.com). Adapters and low-quality data were trimmed using the eautils package fastq-mcf 1.04.636 (https://expressionanalysis.github.io/ea-utils/). For nanopore sequence data, adapter trimming was performed using Porechop v.0.1.0 (https://github.com/rrwick/Porechop). Genome assembly was completed using long reads, with read correction performed with Canu v1.8 (49), followed by assembly in SMARTdenovo github commit id 61cf13d (50). The draft assembly was corrected using the corrected nanopore reads through five rounds of Racon github commit 24e30a9 (51) and then by raw fast5 files using 10 rounds of Nanopolish v0.9.0 (52). Illumina sequencing reads were then used to polish the resulting assembly through 10 rounds of Pilon v1.17 (53). After genome assembly, BUSCO v3 was run to assess evolutionarily conserved gene content (54), using the Saccharomycetales_odb9 gene database. The *Saccharomycetales* database contains 1711 genes, which are therefore expected to be present in *S. stipitis*. Of these, 1,683 (98.36%) were identified in the Y-7124 assembly, demonstrating a good level of completeness (>95%) (see Table S8). Assembly size and contiguity statistics were assessed using QUAST v4.5 (55). This initial assembly of the nuclear genome contained 10 contigs. A chromosome-level assembly was produced by identification of overlapping regions between the contigs: a 244-kbp overlapping region between contigs 7 and 2 led to the final assembly of chromosome 1, and an 83-kbp overlapping region between contigs 9 and 10 led to the final assembly of chromosome 8.

### Genome annotation.

Genome annotation was performed using FUNGAP v1.0.1 (56) with fastq reads from NCBI SRA accession SRR8420582 (BioSample SAMN09064163) used as RNA-Seq training data and protein sequences taken from NCBI assembly accession GCA_000209165.1 for *S. stipitis* NRRL Y-11545 (CBS6054) used, for example, proteins. Protein fasta files were extracted from predicted gene models using the yeast mitochondrial code (code 3) and the alternative yeast nuclear code (code 12). Functional annotation of gene models was performed through BLASTp searches versus all proteins from the NCBI reference fungal genomes (downloaded 11 April 2020), retrieving the top-scoring blast hit with an E value of <1 × 10^−30^. These annotations were supplemented with domain annotations from Interproscan v5.42-78.0 (57). The annotated genome was submitted to the NCBI, with submission files prepared using GAG v2.0.1 (http://genomeannotation.github.io/GAG.), Annie github commit 4bb3980 (http://genomeannotation.github.io/annie), and table2 as n_GFF v1.23.377 (available from https://ftp.ncbi.nih.gov/toolbox/ncbi_tools/converters/by_program/tbl2asn/).

### Comparative genomics.

Whole-genome alignment between Y-7124 and Y-11545 was performed using the nucmer tool from the MUMmer package v4.0 (58), and results were visualized using Circos v0.6 (59). Orthology analysis was performed between predicted proteins from these isolates using OrthoFinder v2.3.11 (60), with results visualized using the package VennDiagram in R (61).

Sequence variants were identified in Y-7124 through comparison to the Y-11545 assembly. Short-read sequence data for Y-7124 were aligned to the reference genome using BWA v0.7.15-r1140 (62) before filtering using using picardtools v2.5.0 to remove optical duplicates (http://broadinstitute.github.io/picard/). SNP and insertion/deletion (InDel) calling were performed using GATK4 (63). Low-confidence variants were then filtered using VCFtools v0.1.15 (64) using minimum mapping quality of 40, a phred quality of 30, a read depth of 10, and a genotype quality of 30. The effect of variants on NRRL Y-11545 gene models was determined using SnpEff v4.2 (65).

### Data availability.

This Whole Genome Shotgun project has been deposited at DDBJ/ENA/GenBank under the accession JADGGA000000000. The version described here is version JADGGA010000000. Illumina and nanopore sequence data associated with this work have been deposited on the Sequence Read Archive (SRA) under BioProject PRJNA609885.
