# Supplementary material for: The Genome of the CTG(Ser1) Yeast Scheffersomyces stipitis Is Plastic
Source: mBio. 2021 Sep 7;12(5):e01871-21. doi: 10.1128/mBio.01871-21 (PMC8546629; doi:10.1128/mBio.01871-21)
Supplement: TABLE S1 [file mbio.01871-21-st001.docx]

**Supplementary Table S1**: List of Strains used in this study

| **Collection** | **Identifier** | **Habitat** | **Region** | **Country** |
| --- | --- | --- | --- | --- |
| NRRL | Y-11545 | Unknown | Delft | The Netherlands |
| NRRL | Y-7124 | Insect (IN) larva on fruit tree | Rhône, Lyon | France |
| NCYC | 1540 | Habitat Insect larva | Rhône, Lyon | France |
| NCYC | 1542 | Insect (IN) larva on fruit tree | Rhône, Lyon | France |
| NCYC | 1566 | Insect (IN) larva on fruit tree | Rhône, Lyon | France |
| NRRL | Y-17104 | Industrial fermentations (IFM), from xylose fermentation | Delft | The Netherlands |
| NRRL | YB-3756 | Pine and coniferous trees (PTR), gymnosperms, dead pine tree, | Gainesville, Florida | USA |
| NRRL | Y-27547 | Insect (IN), beetle, Odontotaenius disjunctus, Passalidae, Burke Co. Shell | Georgia | USA |
| NRRL | Y-27548 | Insect (IN), beetle, Odontotaenius disjunctus, Passalidae, | Lake Herrick Park, Georgia | USA |
| NRRL | Y-27549 | Insect (IN), beetle, Passalidae, | Orangeburg County, South Carolina | USA |
| NRRL | Y-27550 | Insect (IN), beetle, Odontotaenius disjunctus, Passalidae, | Burden, Baton Rouge, Louisiana | USA |
| NRRL | YB-1611 | Hard wood trees (HTR), angiosperms, shagbark hickory, | Peoria, Illinois | USA |
| NRRL | Y-12759 | Soil or rock (SL), forest soil, | Georgia | USA |
| NRRL | YB-3713 | Frass or Insect tunnels (FR), frass on dead oak log, | Gainesville, Florida | USA |
| NRRL | Y-27552 | Insect (IN), beetle, Verres sternbergianus, Passalidae, | Barro Colorado Island | Panama |
| NRRL | Y-27535 | Insect (IN), gut of a Passalid beetle, | Kansas | USA |
| NRRL | Y-8209 | Tree, unknown type (TR), tree trunk, | New Orleans, Louisiana | USA |
| NRRL | Y-8271 | Frass or insect tunnels (FR), frass, | New Orleans, Louisiana | USA |
| NRRL | Y-17100 | Insect (IN), insect larvae on fruit tree, | Rhône, Lyon | France |
| NRRL | Y-27551 | Insect (IN), beetle, Odontotaenius disjunctus, Passalidae | Duches Dr. Park, Baton Rouge, Louisiana | USA |
| NRRL | Y-27555 | Insect (IN), beetle, Odontotaenius disjunctus, Passalidae, | Oxford, Pennsylvania | USA |
| NRRL | YB-1337 | Tree, unknown type (TR), rotted log, | Wohlwend Farm, Marion, Illinois | USA |
| NRRL | YB-2051 | Frass or insect tunnels (FR), frass, dead black oak. | Missouri | USA |
| NRRL | YB-3619 | Frass or insect tunnels (FR), frass, American elm. |  | USA |
| NRRL | Y-27553 | Insect (IN), beetle, Verres sternbergianus, Passalidae, | Barro Colorado Island | Panama |
| NRRL | YB-1762 | Frass or insect tunnels (FR), frass, rotten logs, Brownfield Woods | Illinois | USA |
| NCYC | 1541 | Insect (IN) larva on fruit tree | Rhône, Lyon | France |
