# Supplementary material for: The Genome of the CTG(Ser1) Yeast Scheffersomyces stipitis Is Plastic
Source: mBio. 2021 Sep 7;12(5):e01871-21. doi: 10.1128/mBio.01871-21 (PMC8546629; doi:10.1128/mBio.01871-21)
Supplement: TABLE S6 [file mbio.01871-21-st006.docx]

**Supplementary Table S6:** Identification of *S. stipitis* natural isolates by Sanger sequencing

| **Code** | **Blast result:**  **D1/D2 domain**  **of 26S rDNA gene** | **% identity** | **E-value** |
| --- | --- | --- | --- |
| ATCC 58784 | *Scheffersomyces stipitis* | 99.47 | 0.0 |
| ATCC 58376 | *S. stipitis* | 99.14 | 0.0 |
| ATCC 62970 | *S. stipitis* | 98.63 | 0.0 |
| ATCC 62971 | *S. stipitis* | 99.65 | 0.0 |
| NRRL Y-17104 | *S. stipitis* | 99.65 | 0.0 |
| NRRL YB-3756 | *S. stipitis* | 99.65 | 0.0 |
| NRRL Y-27547 | *S. stipitis* | 99.82 | 0.0 |
| NRRL Y-27548 | *S. stipitis* | 99.65 | 0.0 |
| NRRL Y-27549 | *S. stipitis* | 99.47 | 0.0 |
| NRRL Y-27550 | *S. stipitis* | 99.82 | 0.0 |
| NRRL YB-1611 | *S. stipitis* | 99.82 | 0.0 |
| NRRL Y-12759 | *S. stipitis* | 99.65 | 0.0 |
| NRRL YB-3713 | *S. stipitis* | 99.13 | 0.0 |
| NRRL Y-27552 | *S. stipitis* | 99.65 | 0.0 |
| NRRL Y-27535 | *S. stipitis* | 99.65 | 0.0 |
| NRRL Y-8209 | *S. stipitis* | 99.82 | 0.0 |
| NRRL Y-8271 | *S. stipitis* | 99.47 | 0.0 |
| NRRL Y-11545 | *S. stipitis* | 99.48 | 0.0 |
| NRRL Y-17100 | *S. stipitis* | 99.65 | 0.0 |
| NRRL Y-27551 | *S. stipitis* | 99.47 | 0.0 |
| NRRL Y-27555 | *S. stipitis* | 99.29 | 0.0 |
| NRRL YB-1337 | *S. stipitis* | 99.82 | 0.0 |
| NRRL YB-1762 | *S. stipitis* | 99.65 | 0.0 |
| NRRL YB-2051 | *S. stipitis* | 99.82 | 0.0 |
| NRRL YB-3619 | *S. stipitis* | 99.31 | 0.0 |
| NRRL Y-7124 | *S. stipitis* | 99.65 | 0.0 |
| NRRL Y-27553 | *S. stipitis* | 99.82 | 0.0 |
