# Supplementary material for: The Genome of the CTG(Ser1) Yeast Scheffersomyces stipitis Is Plastic
Source: mBio. 2021 Sep 7;12(5):e01871-21. doi: 10.1128/mBio.01871-21 (PMC8546629; doi:10.1128/mBio.01871-21)
Supplement: TABLE S7 [file mbio.01871-21-st007.docx]

**Supplementary Table S7**: Statistical difference on growth rate, maximum OD and lag time of each natural isolate used in this study respect to the reference strain Y-11545.

| **Growth rate** | | **SC-G** | **SC-X** | **SC-G+X** |
| --- | --- | --- | --- | --- |
|  | *** | Y-27553 YB-3619 Y-27551 Y-8271 Y-8209 Y-12759 | YB-3619  YB-2051  YB-1762  YB-1337 Y-27555  Y-8271  YB-1611 | Y-27553  YB-2051  YB-1762  YB-1337  Y-27555  Y-8271  Y-8209  YB-3713  Y-27550  Y-27549 |
| Higher | ** | Y-27552 | Y-12759  Y-27547 | Y-12759  Y-27548  Y-27547 |
|  | * | YB-1762 Y-27548 Y-27547 | Y-27552  Y-27550  Y-27549  Y-27548 | YB-3619  YB-1611 |
|  | * | Y-17100 | - | Y-7124 |
| Lower | ** | Y-17104 | - | - |
|  | *** | Y-27535 NCYC 1566 | Y-27535 NCYC 1566 | Y-27535  NCYC 1566 NCYC 1541 |
| **Maximum OD** | | **SC-G** | **SC-X** | **SC-G+X** |
|  | *** | - | - | - |
| Higher | ** | - | - | - |
|  | * | - | NCYC 1541 | - |
|  | * | Y-27552 | - | - |
| Lower | ** | Y-7124 | NCYC 1566 | Y-27535 |
|  | *** | NCYC 1566 Y-17104 Y-27535 | Y-27535 | NCYC 1540  NCYC 1541  NCYC 1566  Y-7124 |
| **Lag time** | | **SC-G** | **SC-X** | **SC-G+X** |
|  | *** | NCYC 1541  NCYC 1542  Y-17104 | NCYC 1542  NCYC 1566  YB-3756  Y-27535 | NCYC 1541  NCYC 1542  NCYC 1566 |
| Higher | ** | Y-17100 | Y-17100  YB-2051 | - |
|  | * | YB-3713  Y-27535  Y-27555 | - | NCYC 1540  Y-17100 |
|  | * | Y-7124 | - | - |
| Lower | ** | - | - | - |
|  | *** | - | - | - |
