# Supplementary material for: The Genome of the CTG(Ser1) Yeast Scheffersomyces stipitis Is Plastic
Source: mBio. 2021 Sep 7;12(5):e01871-21. doi: 10.1128/mBio.01871-21 (PMC8546629; doi:10.1128/mBio.01871-21)
Supplement: TABLE S8 [file mbio.01871-21-st008.docx]

**Supplementary Table S8.** Quality assessment of Y-7124 Genome sequencing

| N. of contigs | 10 |
| --- | --- |
| Total length (Mbp) | 15.69 |
| Largest contig (Mbp) | 2.69 |
| N50 (Mbp) | 1.88 |
| N75 (Mbp) | 1.67 |
| L50 | 4 |
| L75 | 6 |
|  |  |
| BUSCO analysis |  |
| Complete | 1683 |
| Duplicated | 13 |
| Fragmented | 11 |
| Missing | 17 |
| Total | 1711 |
