# Supplementary material for: The Genome of the CTG(Ser1) Yeast Scheffersomyces stipitis Is Plastic
Source: mBio. 2021 Sep 7;12(5):e01871-21. doi: 10.1128/mBio.01871-21 (PMC8546629; doi:10.1128/mBio.01871-21)
Supplement: TABLE S9 [file mbio.01871-21-st009.docx]

**Supplementary Table S9**: Summary of the SNPs observed between the natural isolates of *S. stipitis* NRRL Y-11545 and NRRL Y-7124.

| Number of variants | 50,495 |
| --- | --- |
| Variant rate | 1 variant / 306 bases |
| Transitions | 30,655 |
| Transversions | 19,725 |
|  |  |
| Region |  |
| Exon | 21,904 (43.38%) |
| Intergenic | 25,680 (50.86%) |
| Other | 2,911(5.85%) |
|  |  |
| Effects |  |
| Missense | 5,622 (25.62%) |
| Nonsense | 28 (0.13%) |
| Silent (synonymous) | 16,294 (74.25%) |
