# Supplementary figures and images for: The Genome of the CTG(Ser1) Yeast Scheffersomyces stipitis Is Plastic
Source: mBio. 2021 Sep 7;12(5):e01871-21. doi: 10.1128/mBio.01871-21 (PMC8546629; doi:10.1128/mBio.01871-21)

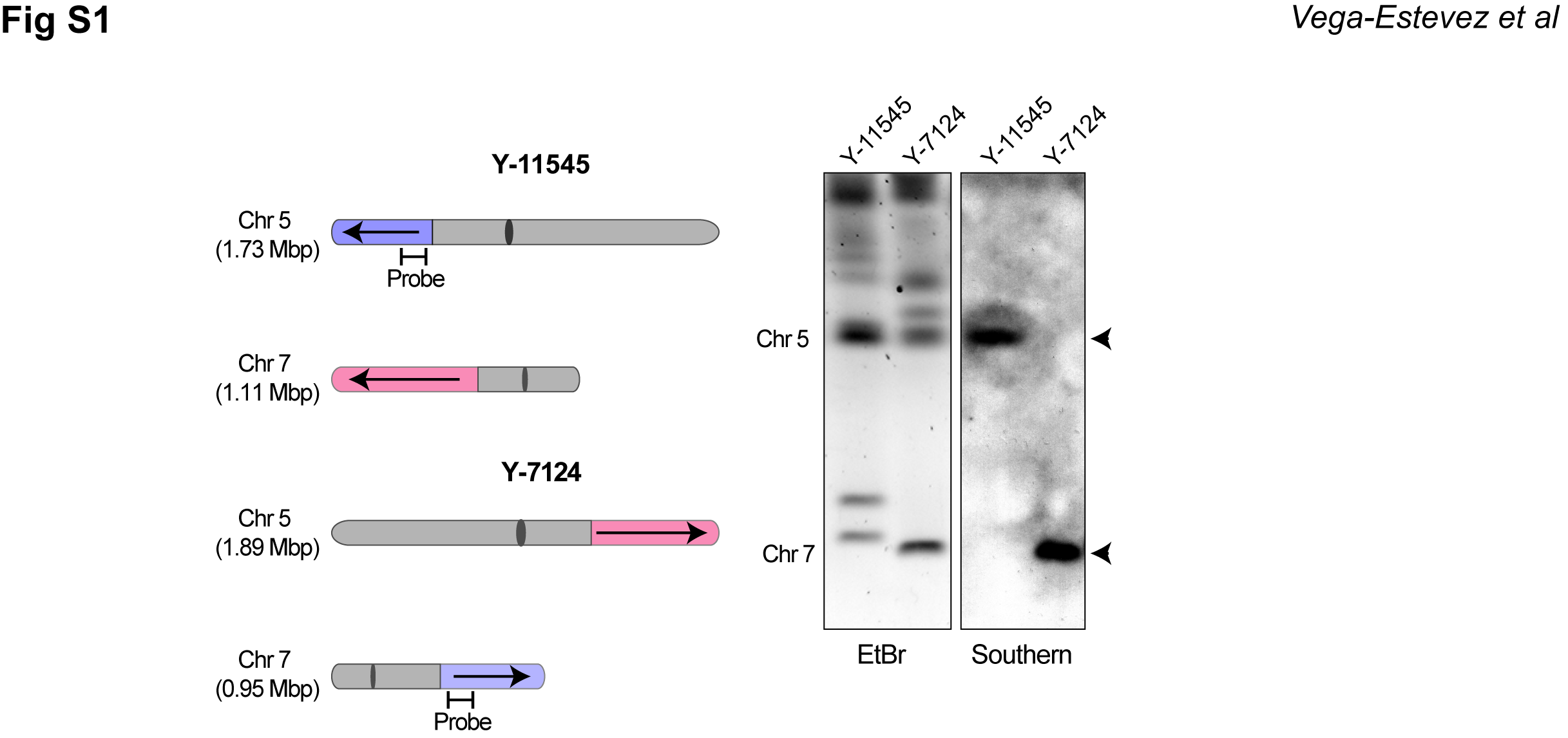

Supplement: FIG S1 [file mbio.01871-21-sf001.tif]
