# Supplementary material for: The Genome of the CTG(Ser1) Yeast Scheffersomyces stipitis Is Plastic
Source: mBio. 2021 Sep 7;12(5):e01871-21. doi: 10.1128/mBio.01871-21 (PMC8546629; doi:10.1128/mBio.01871-21)
Supplement: TABLE S2 [file mbio.01871-21-st002.docx]

**Supplementary Table S2:** Primers used in this study

| NAME | SEQUENCE |
| --- | --- |
| AB798 | GCATATCAATAAGCGGAGGAAAAG |
| AB799 | GGTCCGTGTTTCAAGACGG |
| AB1028 | CAACTTCAAACACCGGCTCG |
| AB1029 | CTGGTGTCGACGGTAAACCA |
